# Supplementary material for: Bio-Organically Acidified Product-Mediated Improvements in Phosphorus Fertilizer Utilization, Uptake and Yielding of Zea mays in Calcareous Soil
Source: Plants (Basel). 2023 Aug 27;12(17):3072. doi: 10.3390/plants12173072 (PMC10490485; doi:10.3390/plants12173072)
Supplement: Supplementary file 1 [file plants-12-03072-s001.zip › plants-2388370-supplementary.pdf]

Table S1. Analysis of variance (Three-Way ANOVA) for determined plant's analysis

Variable	Factor	Type III sum of squares	Degree of freedom	Mean of squares	F value	P value
**Plant Height (cm)**	Acid A	15914.5	2	7957.26	466.93	<0.00001***
	P	4428.7	2	2214.35	129.94	<0.00001***
	PSB	6902.0	1	6902.04	405.01	<0.00001***
	Acid A*P	3054.4	4	763.61	44.81	<0.00001***
	Acid A*PSB	854.1	2	427.04	25.06	<0.00001***
	P*PSB	689.3	2	344.63	20.22	<0.00001***
	AcidA*P*PSB	362.7	4	90.67	5.32	<0.0018**
**Leaf Area (cm^2)**	Acid A	108973	2	54486.3	743.14	<0.00001***
	P	22238	2	11118.9	151.65	<0.00001***
	PSB	82798	1	82798.3	1129.28	<0.00001***
	Acid A*P	18014	4	4503.4	61.42	<0.00001***
	Acid A*PSB	9803	2	4901.3	66.85	<0.00001***
	P*PSB	2403	2	1201.3	16.38	<0.00001***
	AcidA*P*PSB	18506	4	4626.6	63.10	<0.00001***
**Root Length (cm)**	Acid A	3889.2	2	1944.60	117.75	<0.00001***
	P	1572.6	2	786.29	47.61	<0.00001***
	PSB	3109.2	1	3109.17	188.26	<0.00001***
	Acid A*P	401.4	4	100.36	6.08	<0.00001***
	Acid A*PSB	421.3	2	210.63	12.75	<0.00001***
	P*PSB	259.9	2	129.95	7.87	<0.00001***
	AcidA*P*PSB	565.3	4	141.32	8.56	<0.00001***
**Shoot Dry Weight (g)**	Acid A	4693.37	2	2346.69	565.09	<0.00001***
	P	466.45	2	233.23	56.16	<0.00001***
	PSB	1896.30	1	1896.30	456.63	<0.00001***
	Acid A*P	332.30	4	83.07	20.00	<0.00001***
	Acid A*PSB	108.26	2	54.13	13.03	<0.00001***
	P*PSB	111.79	2	55.89	13.46	<0.00001***
	AcidA*P*PSB	310.07	4	77.52	18.67	<0.00001***
**Root Dry Weight (g)**	Acid A	507.18	2	253.588	48.43	<0.00001***
	P	70.12	2	35.060	6.70	0.0034**
	PSB	315.38	1	315.375	60.23	<0.00001***
	Acid A*P	81.35	4	20.338	3.88	0.0101**
	Acid A*PSB	21.58	2	10.792	2.06	0.1421*
	P*PSB	19.53	2	9.764	1.86	0.1696*
	AcidA*P*PSB	21.06	4	5.264	1.01	0.4175*
**Cobs Dry Weight (g)**	Acid A	2396.33		1198.17	45.22	<0.00001**
	P	510.78		255.39	9.64	0.0004***
	PSB	1300.46		1300.46	49.08	<0.00001***
	Acid A*P	152.22		38.06	1.44	0.2420*
	Acid A*PSB	742.26		371.13	14.01	<0.00001***
	P*PSB	214.93		107.46	4.06	0.0258**
	AcidA*P*PSB	125.52		31.38	1.18	0.3342*
**Cob Length (cm)**	Acid A	139.583	2	69.7917	75.37	<0.00001***
	P	10.028	2	5.0139	5.42	0.0088**
	PSB	72.338	1	72.3380	78.12	<0.00001***
	Acid A*P	16.056	4	4.0139	4.33	0.0058**
	Acid A*PSB	17.954	2	8.9769	9.69	0.0004***
	P*PSB	4.843	2	2.4213	2.61	0.0870**

	AcidA*P*PSB	23.574	4	5.8935	6.36	0.0006**
**Cob Diameter (mm)**	Acid A	220.697	2	110.349	238.07	<0.00001***
	P	17.623	2	8.811	19.01	<0.00001***
	PSB	138.560	1	138.560	298.93	<0.00001***
	Acid A*P	8.305	4	2.076	4.48	0.0049**
	Acid A*PSB	53.987	2	26.994	58.24	<0.00001***
	P*PSB	1.734	2	0.867	1.87	0.1688*
	AcidA*P*PSB	12.563	4	3.141	6.78	<0.00001***
**Chlorophyll Content ($mg\ g^{-1}$)**	Acid A	784.06	2	392.032	98.81	0.0004***
	P	40.56	2	20.282	5.11	0.0111**
	PSB	325.12	1	325.116	81.94	<0.00001**
	Acid A*P	96.35	4	24.088	6.07	0.0008***
	Acid A*PSB	159.84	2	79.921	20.14	<0.00001***
	P*PSB	37.01	2	18.505	4.66	0.0158**
	AcidA*P*PSB	76.57	4	19.144	4.82	0.0032**
**Stomatal Conductance ($mmol\ m^{-2}s^{-1}$)**	Acid A	8135.8	2	4067.91	397.23	<0.00001**
	P	1316.7	2	658.37	64.29	<0.00001***
	PSB	2041.2	1	2041.19	199.32	<0.00001***
	Acid A*P	161.0	4	40.24	3.93	0.0095**
	Acid A*PSB	97.1	2	48.57	4.74	0.0148**
	P*PSB	289.6	2	144.81	14.14	<0.00001***
	AcidA*P*PSB	181.6	4	45.41	4.43	0.0051**
**Photosynthetic Rate ($\mu mol\ CO_2\ m^{-2}s^{-1}$)**	Acid A	496.68	2	248.341	88.68	<0.00001**
	P	426.62	2	213.309	76.17	<0.00001***
	PSB	299.86	1	299.862	107.07	<0.00001***
	Acid A*P	128.20	4	32.049	11.44	<0.00001***
	Acid A*PSB	2.42	2	1.210	0.43	0.6524^{ns}
	P*PSB	65.11	2	32.556	11.62	<0.00001***
	AcidA*P*PSB	85.02	4	21.255	7.59	<0.0001**
**Transpirational Rate ($mmol\ m^{-2}s^{-1}$)**	Acid A	19.7288	2	9.8644	59.86	<0.0002**
	P	1.5012	2	0.7506	4.55	0.0172*
	PSB	13.2759	1	13.2759	80.56	<0.00001***
	Acid A*P	0.0985	4	0.0246	0.15	0.9621^{ns}
	Acid A*PSB	0.1572	2	0.0786	0.48	0.6244^{ns}
	P*PSB	0.1896	2	0.0948	0.58	0.5676*
	AcidA*P*PSB	0.3101	4	0.0775	0.47	0.7571^{ns}
**Water Use Efficiency (%)**	Acid A	10.2570	2	5.12850	10.08	<0.0003**
	P	3.1974	2	1.59869	3.14	0.0552*
	PSB	6.8908	1	6.89077	3.55	<0.0008***
	Acid A*P	0.4321	4	0.10804	0.21	0.9298^{ns}
	Acid A*PSB	6.7565	2	3.37827	6.64	0.0035**
	P*PSB	0.6945	2	0.34725	0.68	0.5116*
	AcidA*P*PSB	0.4714	4	0.11786	0.23	0.9187^{ns}
**Shoot P (%)**	Acid A	0.22305	2	0.11153	209.71	<0.00001**
	P	0.01291	2	0.00645	12.14	0.0001***
	PSB	0.09463	1	0.09463	177.93	<0.00001***
	Acid A*P	0.00069	4	0.00017	0.32	0.8613^{ns}
	Acid A*PSB	0.00097	2	0.00048	0.91	0.4126*
	P*PSB	0.00095	2	0.00048	0.90	0.4167*
	AcidA*P*PSB	0.00342	4	0.00086	1.61	0.1932*
**Root P (%)**	Acid A	0.10931	2	0.05465	105.19	<0.00001***
	P	0.01030	2	0.00515	9.91	<0.0004***

	PSB	0.05273	1	0.05273	101.50	<0.00001***
	Acid A*P	0.00413	4	0.00103	1.99	0.1170*
	Acid A*PSB	0.00678	2	0.00339	6.52	0.0038**
	P*PSB	0.00225	2	0.00113	2.17	0.1293*
	AcidA*P*PSB	0.00298	4	0.00075	1.43	0.2424*
**Grain P (%)**	Acid A	0.18685	2	0.09343	116.65	<0.00001***
	P	0.01527	2	0.00764	9.53	<0.0005***
	PSB	0.05967	1	0.05967	74.50	<0.00001***
	Acid A*P	0.00519	4	0.00130	1.62	0.1908*
	Acid A*PSB	0.02153	2	0.01076	13.44	<0.00001***
	P*PSB	0.00055	2	0.00027	0.34	0.7125^{ns}
	AcidA*P*PSB	0.00922	4	0.00230	2.88	0.0364**
**Crude Fat (%)**	Acid A	0.84487	2	0.42244	246.81	<0.00001***
	P	0.18828	2	0.09414	55.00	<0.00001***
	PSB	0.39956	1	0.39956	233.44	<0.00001***
	Acid A*P	0.02219	4	0.00555	3.24	0.0227**
	Acid A*PSB	0.00558	2	0.00279	1.63	0.2098*
	P*PSB	0.00842	2	0.00421	2.46	0.0998*
	AcidA*P*PSB	0.02716	4	0.00679	3.97	0.0091**
**Crude Fiber (%)**	Acid A	6.33427	2	3.16714	315.33	<0.00001***
	P	0.92280	2	0.46140	45.94	<0.00001***
	PSB	1.05728	1	1.05728	105.27	<0.00001***
	Acid A*P	0.12090	4	0.03022	3.01	0.0306**
	Acid A*PSB	0.08224	2	0.04112	4.09	0.0250**
	P*PSB	0.08316	2	0.04158	4.14	0.0241**
	AcidA*P*PSB	0.11259	4	0.02815	2.80	0.0401**
**Crude Protein (%)**	Acid A	20.8182	2	10.4091	157.42	<0.00001***
	P	9.1463	2	4.5731	69.16	<0.00001***
	PSB	8.0813	1	8.0813	122.22	<0.00001***
	Acid A*P	0.8894	4	0.2224	3.36	0.0194**
	Acid A*PSB	0.0467	2	0.0234	0.35	0.7047^{ns}
	P*PSB	1.1303	2	0.5651	8.55	0.0009***
	AcidA*P*PSB	0.5759	4	0.1440	2.18	0.0912*
**Ash Contents (%)**	Acid A	1.99339	2	0.99669	241.76	<0.00001***
	P	0.77957	2	0.38979	94.55	<0.00001***
	PSB	0.47508	1	0.47508	115.24	<0.00001***
	Acid A*P	0.05655	4	0.01414	3.43	0.0179**
	Acid A*PSB	0.06040	2	0.03020	7.33	0.0021**
	P*PSB	0.00557	2	0.00279	0.68	0.5150^{ns}
	AcidA*P*PSB	0.03762	4	0.00940	2.28	0.0795*
**Thousand Grains Weight** (g)	Acid A	157387	2	78693.4	978.06	<0.00001***
	P	16477	2	8238.6	102.40	<0.00001***
	PSB	31877	1	31876.7	396.19	<0.00001***
	Acid A*P	11380	4	2845.1	35.36	<0.00001***
	Acid A*PSB	1494	2	746.8	9.28	0.0006**
	P*PSB	8542	2	4270.8	53.08	<0.00001***
	AcidA*P*PSB	1272	4	318.1	3.95	0.0092**

Acid A: Acidified organic amendments; P: phosphorus sources; PSB: phosphorus solubilizing bacteria; Acid A ×P: Acidified organic amendments × phosphorus; Acid A ×PSB: Acidified organic amendments × phosphorus solubilizing bacteria; P×PSB: phosphorus × phosphorus solubilizing bacteria; Acid A ×P×PSB: Acidified organic amendments × phosphorus × phosphorus solubilizing bacteria. Whereas ***: P<0.0001; **: P<0.05; *: P>0.05; ns: not significant.
